# Microbiome analysis and epigenetic patterns revealed distinct differences between two elm species with contrasting Dutch elm disease resistance

**DOI:** 10.1186/s40168-026-02488-5

**Published:** 2026-08-19

**Authors:** Hans Hoenicka, Kristina Ulrich, Charlotte Haffner, Marta Starczak, Daniel Gackowski, Ben Bubner, Andreas Ulrich

**Affiliations:** 1 Thünen-Institute of Forest Genetics, Sieker Landstr. 2, Großhansdorf, 22927 Germany; 2 Thünen Institute of Forest Genetics, Eberswalder Chaussee 3a, Waldsieversdorf, 15377 Germany; 3 Department of Clinical Biochemistry, Faculty of Pharmacy, Collegium Medicum in Bydgoszcz, Nicolaus Copernicus University in Toruń, Karlowicza 24, Bydgoszcz, 85-095 Poland; 4 Microbial Biogeochemistry, Leibniz Centre for Agricultural Landscape Research (ZALF), Eberswalder Straße 84, Müncheberg, 15374 Germany

**Keywords:** Dutch elm disease, Microbiome, Bacterial and fungal communities, Lichens, Algae, Epigenetics, *Ulmus*, Stress resistance, Forest trees

## Abstract

**Background:**

Dutch elm disease (DED), caused by the fungus *Ophiostoma novo-ulmi*, has devastated elm species in Europe and North America for over a century. While the Chinese elm (*Ulmus parvifolia*) frequently displays notable resistance to DED, the European wych elm (*Ulmus glabra*) remains highly susceptible. Understanding microbiome interaction and epigenetic factors might help to explain this contrasting resilience and subsequently improve disease management.

**Results:**

Seedlings of both elm species were germinated and maintained under identical environmental conditions. Comparative metabarcoding revealed distinct differences in bacterial and eukaryotic microbiomes, as well as in their associations with lichenized fungi and their photobiont algae. In *U. parvifolia*, 24 eukaryotic and 14 bacterial taxa were identified as the most differentially abundant microbiome components, and exhibited significantly different levels compared to *U. glabra*. In particular, *Leotiomycetes* fungi and *Rhizobiales* bacteria, both previously implicated in resistance to pathogens, were strongly enriched in *U. parvifolia*. Lichenized fungi and chlorophyte algae were likewise more abundant in *U. parvifolia*. In *U. glabra*, 11 eukaryotic and 16 bacterial taxa were significantly more abundant, including *Nocardioides* bacteria and *Leptospora* fungi.

Ultra-performance liquid chromatography coupled with mass spectrometry revealed that, compared to *U. glabra*, *U. parvifolia* exhibited higher levels of 5-(hydroxymethyl)-2′-deoxycytidine and lower levels of 5-methyl-2′-deoxycytidine and N6-methyl-2′-deoxyadenosine in DNA, as well as increased amounts of 5-methylcytidine, N6-methyladenosine, and 5-hydroxymethyluridine in RNA. The elevated levels of well-established epigenetic markers in DNA and RNA are remarkable. However, it remains unclear what part of the holobiont may be responsible for this effect.

**Conclusions:**

The contrasting susceptibility of Chinese and European elms to DED correlates with pronounced differences in their associated microbial communities and lichens, as well as in patterns of epigenetic nucleoside modifications. In particular, the enrichment of specific stress-associated microbiome components, together with lichen associations and elevated levels of modified DNA and RNA nucleosides in *U. parvifolia*, may underlie its increased tolerance to DED. Although this study did not identify the factors responsible for DED resistance, it provides valuable insights into microbiomic and epigenetic features that could contribute to the development of new approaches to combat DED.

Video Abstract

**Supplementary Information:**

The online version contains supplementary material available at 10.1186/s40168-026-02488-5.

## Background

In the last century, forests worldwide have been increasingly affected by the invasion of non-native forest pathogens. These organisms have caused significant harm, resulting in substantial economic losses and drastic environmental changes. The primary driver of pathogen introduction and distribution is the expansion of international trade, which allows these alien species to exceed their natural distribution ranges [1]. Furthermore, global warming directly impacts pathogens by creating favorable conditions for their propagation and establishment in areas that were previously unsuitable. As a result, many tree species are now confronted with entirely new biotic and abiotic stress factors.

Dutch elm disease (DED) is one of the most striking examples of an invasive non-native tree pathogen. It is caused by the fungi *Ophiostoma ulmi, Ophiostoma himal-ulmi*, and *Ophiostoma novo-ulmi*; the latter has led to widespread mortality among elm species, including wych elm (*Ulmus glabra*) and field elm (*Ulmus minor*), across Europe [2]. The disease disrupts water transport within the xylem, causing wilting and ultimately death due to the loss of water conductivity [2, 3].

Despite over a hundred years of extensive research into resistance and control methods for DED, very little progress has been made. Consequently, as a result of these devastating epidemics, elms have largely lost their ecological role in European forest ecosystems and urban landscapes [4]. Once keystone species in many European forests, elms are now significantly reduced in number and size, leading to alterations in forest composition and urban greenery across the continent [2]. Despite a report on potentially resistant field elms [5], the lack of resistant clones has been the main hindrance to resistance breeding against DED and other tree pathogens. Furthermore, the long time required for elms and other tree species to reach sexual maturity makes it more difficult to induce resistance through traditional breeding techniques, compared to annual crops [6, 7]. While genetic modification and genome editing present viable options for quickly developing resistance in trees, these methods are still restricted in practical applications in numerous countries [8]. Therefore, it is crucial to investigate alternative approaches, e.g., based on microbiome [9–14] or epigenome [15–18] optimization for a faster and more effective induction of stress resistance in tree species.

Several Asiatic elm species, such as the Chinese elm (*Ulmus parvifolia*), exhibit a high level of resistance to DED [19], likely due to a co-evolutionary relationship with the fungi that promote this illness. However, the underlying mechanisms of this resistance (potentially involving the genome, epigenome, and/or microbiome) remain largely unknown. Comparative studies with DED-resistant and susceptible clones and elm species represent a promising strategy against this illness. To date, most studies on DED have focused on the genetic level [2, 4, 20–22], while no epigenetic studies specifically related to DED have been conducted so far. Some reports are available on the mycobiome of elms [14, 23–25], but none exist regarding other parts of the microbiome, such as the bacterial or algal communities. The relative abundance of two orders of endophytic fungal yeasts (*Chaetothyriales* and *Cystobasidiales*) has been linked to the resistance of certain field elm genotypes against DED [14]. Additionally, three families of yeasts (*Buckleyzymaceae*, *Trichomeriaceae*, and *Bulleraceae*) have been associated with field elms that exhibit resistance to DED [14]. This correlation suggests that these specific fungi may play a role in enhancing the trees′ defense mechanisms.

Stress resistance arises from complex interactions among plants, their microbiomes, genetic and epigenetic factors, and the environment [26]. Certain microbial-derived compounds of low-molecular-weight could potentially act as factors influencing genomic and epigenomic mechanisms in both the host organism and its associated microbiome [27]. Microbes produce various low-molecular-weight molecules, including methyl and acetyl groups, enzymes like phosphatases and methyltransferases, as well as biotin and kinases similar to those found in eukaryotes [28]. These microbial products may directly influence epigenetic mechanisms and stress resistance in eukaryotic organisms [29].

In this study, we hypothesized that the resistance of *U. parvifolia* to DED might not only be due to genetic differences but also to the microbiome composition or epigenetic factors. A key prerequisite for these effects is the presence of significant differences between susceptible and resistant elm species. Accordingly, in this study, we compared the microbiome and epigenetic variation in coeval seedlings of *U. glabra* and *U. parvifolia* grown under shared environmental conditions.

## Material and methods

### Plant material

Wych elm seeds were obtained in spring 2020 from the Forest Gene Resources Center (FOGZ) in Lippstadt, Germany, while Chinese elm seeds were sourced in fall 2019 from the Arboretum Lohbrügge in Hamburg, Germany. Chinese Elm seeds were stratified until spring 2020, whereas wych elm seeds were sown directly after collection in the same year. The plants were germinated and grown together in the spring of 2020 in the greenhouse of the Institute of Forest Genetics in Grosshansdorf, Germany (53° 39′ 42.5952″ N, 10° 15′ 12.7764″ E), using the same soil mixture and pots for all plants. Subsequently, a field trial was established nearby (53° 40′ 19.70′′ N, 10° 16′ 19″ E) using these plants. Plant material for epigenetic and microbiome studies was obtained in June 2023 from seedlings in the field trial. Twelve biological replicates were studied for each elm species. Elm stems were collected from these seedlings for microbiome analyses, while leaves were collected for epigenetic studies. Both types of samples were stored at − 20 °C until further processing.

### Microbiome analyses

#### DNA extraction and amplicon sequencing from elm cortex and cambium tissues

Metabarcoding analysis was conducted using surface-sterilized stems from which samples containing cortex and cambium tissues were carefully collected under sterile conditions (supplementary information). Between 50 and 100 mg of plant material was used for DNA isolation following Bruegmann et al. [30], with some modifications. The DNA purity (OD260/OD280 and OD260/OD230 ratios) was maintained between 1.8 and 2.1, and the concentration was measured using a NanoDrop spectrophotometer and a Qubit fluorometer. The bacterial community was analysed by 16S rRNA gene amplicon sequencing using the primers 799 F and 1115R, which exclude the chloroplast and mitochondrial DNA of the host plant [31, 32]. PCR amplification of the DNA samples was performed as described by Ulrich et al. [33]. Negative controls consisted of one extraction blank per extraction batch and 1–2 PCR no-template controls (NTCs) per PCR. The absence of amplification in negative controls indicated that contamination was negligible. To study the fungal community, the ITS2 region was amplified with the primers fITS7 [34] and ITS4 [35], preventing the amplification of the host plant DNA. PCR amplification was conducted as described by Ihrmark et al. [34]. The primers were extended with a heterogeneity spacer and a barcode sequence. The amplicons were checked with gel electrophoresis and mixed to equimolar DNA concentrations. The library preparation and Illumina MiSeq 300-bp paired-end sequencing were performed at LGC Genomics Berlin (Berlin, Germany).

#### Data processing and microbiome analysis

Demultiplexing and clipping of barcode and primer sequences were conducted by bcl2fastq v2.20 (Illumina, 2024). The paired sequence reads were processed with the DADA2 R package v. 1.22 [36]. The algorithm was applied for quality filtering, denoising and removing chimeric sequences. The amplicon sequence variants (ASVs) provided by DADA2 pipeline were taxonomically assigned by the naive Bayesian classifier method using the RDP reference database training set 18 or the UNITE ITS database v.9.0 (2023–07–25). In addition, the assignment was completed at the species level if the ASVs exactly matched the sequence of the reference strain of only one species. The paired sequence reads were deposited in the public repository Sequence Read Archive with the accession number PRJNA1338362.

Statistical analyses were performed using the phyloseq, vegan, plyr, dplyr, Biostrings, ape and ggplot2 packages of R 4.2.2 (R Core Team, 2023) as well as MicrobiomeAnalyst [37]. After evaluation of the reads, three samples (P04, P05 and G01) from the elm trees were removed due to low read numbers. All samples were rarefied to the minimum number of sequences among all samples. Specifically, the bacterial dataset was rarefied to 21,584 sequences per sample, and the fungal dataset to 19,739 sequences per sample. Alpha diversity indices such as richness, Chao 1, Shannon, and Simpson were calculated. Microbiome composition was compared using principal coordinate analysis (PCoA) based on a Bray–Curtis distance matrix of the ASVs. Significant differences in community structure were tested using analysis of similarity (ANOSIM). Multivariate homogeneity of group dispersions (variances) was analyzed using betadisper. Differential abundance analysis between the microbiomes of the two elm species was calculated at different taxonomic levels using EdgeR’s generalized linear model approach with the false discovery rate (FDR) for multiple test correction [38]. The core microbiome was determined at the genus level and defined as comprising those taxa that occurred with a relative abundance of > 0.2% in at least 70% of the respective samples. To visualize differences in the core microbiomes, the data were used for a taxonomic network, constructed using Cytoscape v3.10.1 [39]. Co-occurrence networks were first calculated based on ASVs present in at least 6 of the 11 replicates using the Molecular Ecological Network Analysis Pipeline [40]. After threshold scanning through the random matrix theory (RMT)-based approach, the networks were separately constructed for the fungal and the bacterial microbiome of *U. parvifolia* and *U. glabra* with an identical similarity threshold 0.77. The topological role of the nodes was assessed by the parameters within-module connectivity (zi) and among-module connectivity (Pi). Connectors (zi ≤ 2.5, Pi > 0.62) are highly linked to several modules and module hubs (zi > 2.5, Pi ≤ 0.62) are highly connected to many nodes in their own modules whereas network hubs (zi > 2.5, Pi > 0.62) acts as both module hubs and connectors [40]. The network was visualized using Cytoscape. The second co-occurrence network was calculated at the genus level with a combined dataset of the eukaryotic and the bacterial microbiome from both elm species using Sparse Estimation of Correlations among Microbiomes (SECOM). It provides two measures of correlations between a pair of taxa, one for linear relationships and the other for nonlinear relationships using the concept of distance correlations [41]. Highly significant correlations (*p* < 0.001) were used for generating and analyzing a network by Cytoscape. The hub taxa were identified as those genera (nodes) in the network that were more central according to their degree (number of associations per node), betweenness centrality (fraction of shortest paths the node is on) and closeness centrality (1/[(distance to all other nodes]), as described by Manirajan et al. [42].

### Epigenetic analyses

#### DNA/RNA isolation and hydrolysis to nucleosides from elm leaves

DNA from leaves was isolated with the GeneMATRIX Plant & Fungi DNA Purification Kit (EURx) and the Lyse CT buffer following the manufacturer’s instructions. RNA isolation was carried out with the GeneMATRIX Universal RNA Purification Kit (EURx) and Lyse Buffer PVP. Nucleic acids were enzymatically hydrolyzed to ribo- and deoxynucleosides using nuclease P1 and shrimp alkaline phosphatase (New England Biolabs) according to a modified protocol described previously [17, 43].

#### Mass spectrometric quantification of non-canonical nucleosides in DNA and RNA

Quantification of non-canonical deoxynucleosides in DNA and ribonucleosides in RNA was performed using isotope-dilution ultra-performance liquid chromatography coupled with tandem mass spectrometry (UPLC–MS/MS), as described in detail by Starczak et al. [43] and Hoenicka et al. [17]. Stable isotope-labeled internal standards were added to all samples prior to analysis. All samples were analyzed in three to five technical replicates, and the technical mean was used for further calculation. The quantities of canonical deoxynucleosides, ribonucleosides and 5-methyl-2′-deoxycytidine (5-mdC) were determined by UV detection. Total deoxynucleosides amount (dN) calculated as doubled sum of 2′-deoxythymidine (dT) and 2′-deoxyguanosine (dG) was used as a reference for quantitative expression of modified ones. Total ribonucleosides amount (rN), calculated as the sum of uridine (rU), guanosine (rG), adenosine (rA) and cytidine (rC) (rU + rG + rA + rC), was used as a reference for quantitative expression of non-canonical ribonucleosides in RNA.

#### Statistical data analysis

Data were analyzed using MaxStat 3.60 software. Data distribution (Kolmogorov–Smirnov test) and variance (Bartlett test) were evaluated, and Welch’s *t*-tests were used for comparing results obtained with both elm species. Significant differences (*p* < 0.05) are indicated in figures; *p* values (*p < 0.05, **p < 0.01, and ***p < 0.001) indicate significant differences between the two elm species.

## Results

### Comparison of the bacterial microbiomes in *U. parvifolia* and *U. glabra* seedlings

The bacterial microbiome of elm stems was characterized by amplicon sequencing targeting the V5–V6 region of the 16S rRNA gene. Plant material was washed and surface-sterilized to target the endosphere; however, due to methodological limitations, some epiphytic bacteria were likely also recovered.

**Fig. 1:**
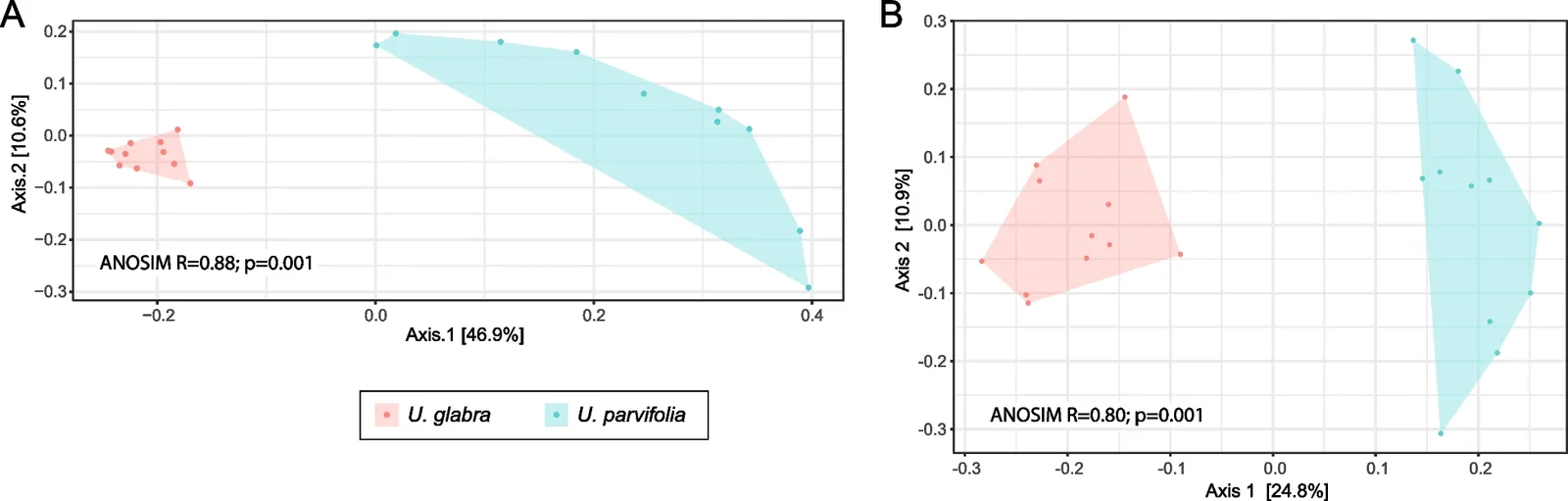
PCoA ordination plot illustrating differences in the bacterial (**A**) and fungal (**B**) communities associated with *U. parvifolia* and *U. glabra* seedlings. Principal coordinate analysis (PCoA) was applied based on the Bray–Curtis distance matrix. Note that the primers fITS7 and ITS4, designed to target the fungal community, also co-amplified algae and the fungal partners of lichens. Moreover, lichens are considered distinct associations rather than part of the plant microbiome

A total of 1.719.717 high-quality reads yielded 699 ASVs. Alpha diversity analysis revealed significantly higher richness (observed ASVs) in *U. glabra* compared with *U. parvifolia* (267 ± 41.0 vs. 153 ± 49.2; mean ± SD; *p* = 0.0006), a pattern confirmed by Shannon diversity indices (4.0 ± 0.16 vs. 3.4 ± 0.49; *p* = 0.002). Principal coordinates analysis (PCoA) revealed clear separation of bacterial communities between the two species (Fig. 1A), supported by ANOSIM (*R* = 0.88, *p* = 0.001). Replicates of *U. parvifolia* seedlings showed significantly greater within-group heterogeneity (group dispersion) (*p* < 0.05).

Taxonomic assignment identified nine bacterial phyla in addition to unclassified taxa. *Pseudomonadota* dominated the communities (54% of total sequences), with *Alphaproteobacteria* accounting for the majority (46.4%), followed by *Betaproteobacteria* (5.5%) and *Gammaproteobacteria* (1.2%). Other major phyla included *Actinomycetota* (35%), *Deinococcota* (6.3%), and *Bacteroidota* (4%), whereas *Firmicutes* accounted for only 0.02% of sequences.

At the genus level, 171 taxa were detected. The most abundant genera were *Methylobacterium* (21.7%), *Sphingomonas* (8.8%), *Klenkia* (6.4%), and *Deinococcus* (6.3%), followed by *Microlunatus* (5.8%), unclassified *Rhizobiales* (5.7%), *Kineococcus* (5.0%), and *Roseomonas* (4.8%) (Supplementary Fig. S1; Supplementary Tables S1–S2).

Differential abundance analysis at the genus level identified 14 taxa (relative abundance > 0.2%) significantly enriched in *U. parvifolia*, together representing 36.9% of the total community. These included unclass. *Rhizobiales* (11.9% relative abundance; 249-fold enrichment), *Frondihabitans* (2.4%; 56-fold), unclass. *Acetobacteraceae* (4.0%; 31-fold), and *Quadrisphaera* (1.5%; 20-fold). Additional genera—including *Microlunatus*, *Hymenobacter*, unclass. *δ-Proteobacteria*, *Amnibacterium*, unclass. *Sphingobacteriaceae*, *Pseudomonas*, and *Rhodococcus*—exhibited 2- to 7-fold increases. In *U. glabra*, 16 genera were significantly enriched (17.4% of the community), including *Nocardioides*, *Aureimonas*, unclass. *Microbacteriaceae*, and *Rhizobium*.

**Fig. 2:**
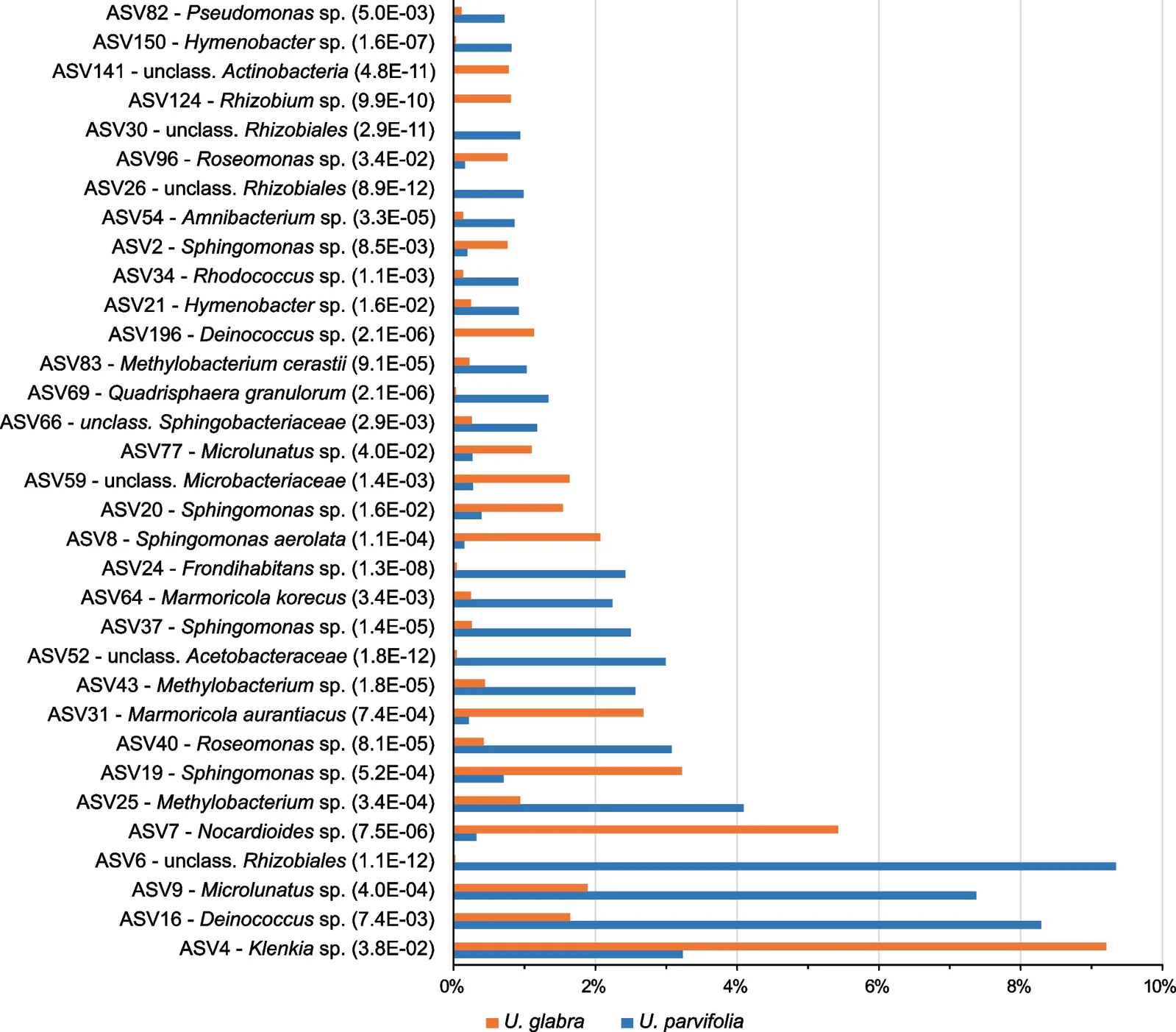
Overview of significantly increased bacterial ASVs in DED-tolerant *U. parvifolia* and susceptible *U. glabra* seedlings, respectively. The ASVs are arranged according their abundance

At the ASV level, 33 ASVs (> 0.4% total abundance) were significantly enriched, of which 20 were associated with *U. parvifolia* (Fig. 2; Supplementary Table S5). Several unclass. *Rhizobiales* ASVs showed the strongest enrichment, with fold changes ranging from 98- to 444-fold relative to *U. glabra*. Additional enriched ASVs in *U. parvifolia* included ASV16 (*Deinococcus* sp.), ASV9 (*Microlunatus* sp.), ASVs 25, 43, and 83 (*Methylobacterium*), ASVs 21 and 150 (*Hymenobacter*), ASV24 (*Frondihabitans*), and ASV52 (unclass. Acetobacteraceae), with fold changes ranging from 4- to 64-fold. The lowest FDR values were observed for ASVs affiliated with unclass. *Rhizobiales*, *Acetobacteraceae*, *Frondihabitans*, and *Hymenobacter*. In *U. glabra*, enriched ASVs included ASV4 (*Klenkia*), ASV7 (*Nocardioides* sp.), ASV31 (*Marmoricola aurantiacus*), and four *Sphingomonas* ASVs.

**Fig. 3:**
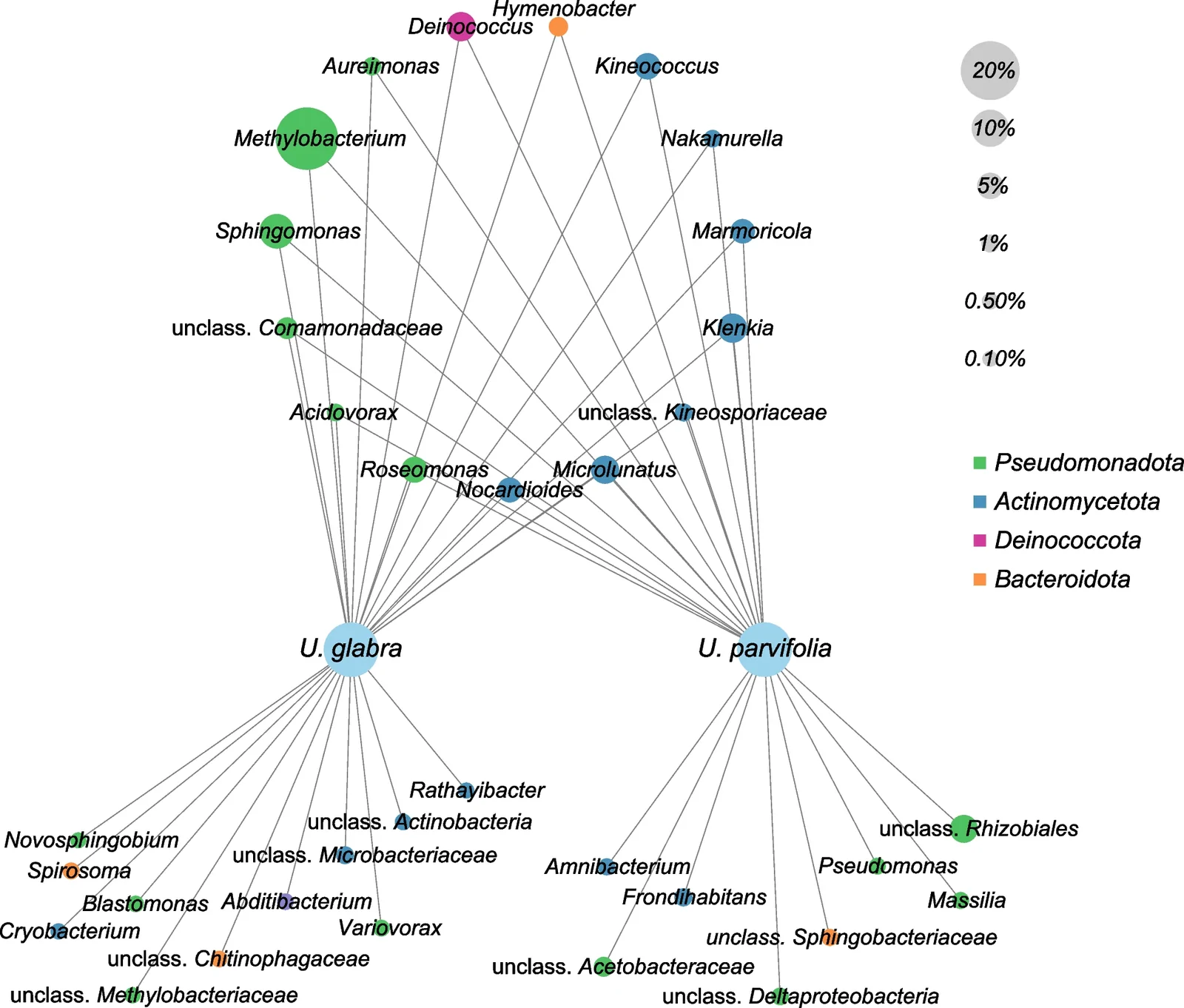
Bacterial core microbiomes of *U. parvifolia* and *U. glabra* seedlings at the genus level. The core microbiomes of both species (taxa occurring in 70% of all replicates of each group that exhibited at least 0.2% relative abundance in the community) were combined for the network analysis. The size of nodes corresponds to the relative abundance in the whole dataset, node labels display their taxonomic affiliation, and the color of the nodes indicates the respective phylum

Core microbiome analysis identified 23 genera (representing 93% of total sequences) in *U. parvifolia* and 26 genera (90%) in *U. glabra*. Fifteen genera were shared between species, including *Methylobacterium* and *Sphingomonas. U. parvifolia* harbored 8 species-specific core genera from three phyla, dominated by unclass. *Rhizobiales*, *Acetobacteraceae*, and *Frondihabitans*, whereas *U. glabra* contained 11 species-specific core genera from four phyla (Fig. 3).

### Eukaryotic microbiome analysis of *U. parvifolia* and *U. glabra* seedlings

The eukaryotic community of the elm microbiome was analyzed using the primers fITS7 [34] and ITS4 [35], targeting the ITS2 region. In addition to fungi, this primer set amplified sequences from green algae of the class *Trebouxiophyceae*, as well as fungi and algae involved in lichen associations. Lichens are symbiotic organisms that can develop macroscopic structures and are therefore typically not considered part of the microbiome. However, early lichen developmental stages are microscopic, and both algae and lichen-associated taxa may play important roles in stress tolerance and host–microbe interactions. These sequence types were therefore retained and considered part of the eukaryotic microbiome in subsequent analyses.

In contrast to the bacterial microbiome, which showed lower alpha diversity in *U. parvifolia*, the eukaryotic microbiome exhibited significantly higher alpha diversity in *U. parvifolia* than in *U. glabra*, as indicated by both ASV richness (193 ± 32.2 vs. 145 ± 41.4; mean ± SD; *p* = 0.016) and the Shannon index (3.3 ± 0.40 vs. 2.7 ± 0.30; *p* = 0.001). Principal coordinates analysis (PCoA; Fig. 1B) revealed clear differences in community structure between the two elm species, which were confirmed by beta diversity analysis (ANOSIM R = 0.80, *p* = 0.001).

At the phylum level, *Ascomycota* dominated the mycobiome of both elm species (82.2% of sequences), followed by *Basidiomycota* (5.9%), while only 0.61% of fungal sequences remained unclassified. *Chlorophyta*, representing green algae inhabiting marine, freshwater and terrestrial environments, accounted for 11.3% of all sequences. At the class level, *Dothideomycetes* were most abundant (60.1%), followed by *Leotiomycetes* (9.18%). At the genus level, *Cladosporium* was most abundant (21.6%), followed by *Elsinoë* (11.05%) and unclass. *Didymellaceae* (9.86%).

At the genus level, the eukaryotic microbiome of *U. parvifolia* was more diverse than that of *U. glabra* (Supplementary Fig. S2; Supplementary Tables S3–S6). In *U. parvifolia*, 21 fungal genera were significantly enriched compared to *U. glabra*, together representing 32.7% of the total community. These included unclass. *Leotiomycetes* (15.8%), *Lembosiniella* (0.4%), *Trichomerium* (1.2%), unclass. *Mycosphaerellales* (0.3%), *Erythrobasidium* (0.4%), *Filobasidium* (2.0%) and *Extremus* (1.0%). In contrast, only 11 genera were significantly enriched in *U. glabra*, yet these accounted for approximately 70% of the community, including *Cladosporium* (32.3%), *Elsinoë* (18.0%), unclass. *Phaeomoniellales* (8.6%), *Diaporthe* (3.5%), *Leptospora* (2.1%) and *Tricellula* (1.2%).

Chlorophyte algae accounted for 16% of the relative abundance in *U. parvifolia* and 6.7% in *U. glabra*. The most abundant algal genus was *Trebouxia* (*U. parvifolia*: 11.8%; *U. glabra*: 6%). *U. parvifolia* showed significantly higher relative abundances of *Pseudochlorella* (2.1%), *Raphidonema* (0.3%), and *Tetratostichococcus* (0.2%), whereas no algal genera were significantly enriched in *U. glabra*.

Ten lichen-associated genera were detected in both elm species (Supplementary Fig. S2; Supplementary Tables S3–S4), accounting for 4% of the relative abundance in *U. parvifolia* and 0.1% in *U. glabra*. *U. parvifolia* showed significantly higher relative abundances of six lichen genera, including *Lecania* (3.4%), *Physcia* (0.4%) and *Bacidia* (0.3%).

**Fig. 4:**
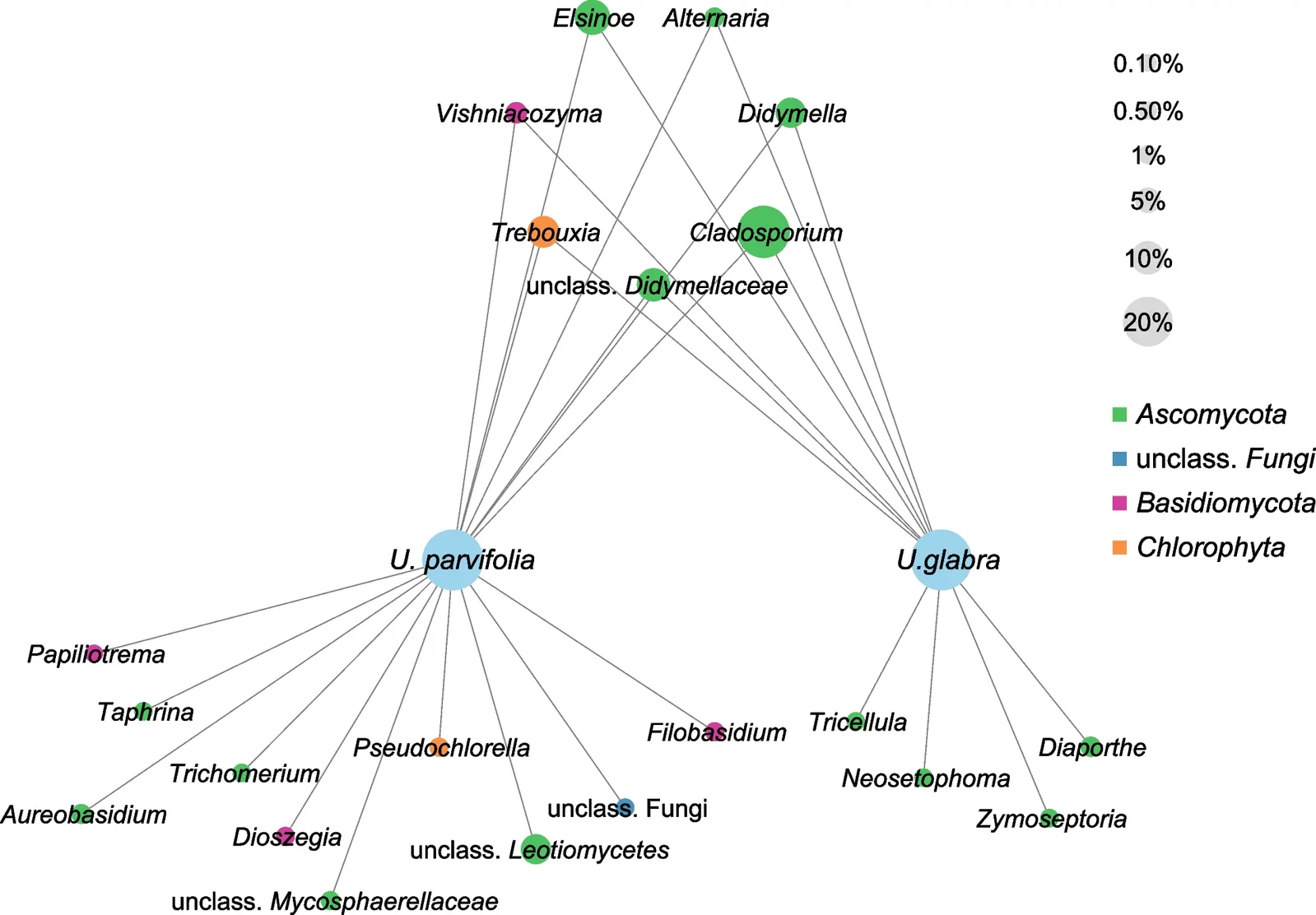
Core eukaryotic microbiome in *U. parvifolia* and *U. glabra* seedlings at the genus level. Genera occurring in 70% of all replicates within each group and exhibiting a minimum relative abundance of 0.2% were summarized as core microbiome and included in the network analysis. Node size corresponds to the relative abundance in the entire dataset, node labels indicate taxonomic affiliation, and node color denotes the respective phylum

The eukaryotic core microbiome of *U. parvifolia* comprised 17 genera representing 81% of all sequences, whereas *U. glabra* contained 11 core genera representing 82% of all sequences (Fig. 4). Seven genera were shared between the two species, including the dominant genera *Cladosporium* and *Elsinoë*. *U. parvifolia* possessed 10 species-specific core genera, with unclass. *Leotiomycetes* accounting for the highest relative abundance (15.8%). These species-specific taxa also included several yeast-like fungi (*Aureobasidium, Papiliotrema, Filobasidium, Dioszegia*, and *Taphrina*) and the green alga *Pseudochlorella*. In contrast, *U. glabra* harboured four species-specific core genera: *Tricellula, Diaporthe, Zymoseptoria*, and *Neosetophoma* (Fig. 4).

**Fig. 5:**
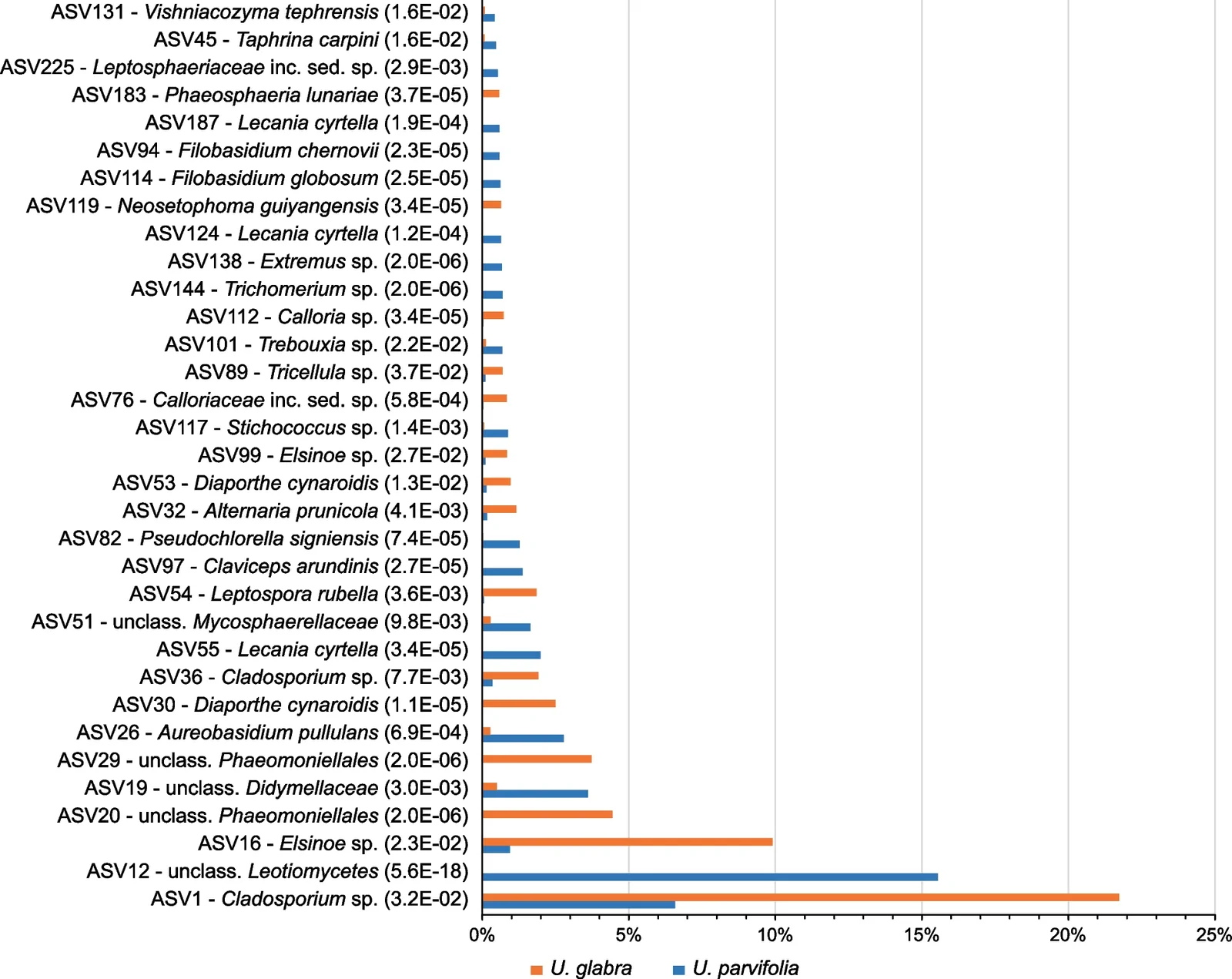
Overview of eukaryotic ASVs that are significantly increased in DED-tolerant *U. parvifolia* and susceptible *U. glabra* seedlings, respectively. The ASVs are arranged according to their total abundance

At the ASV level, differential abundance analysis identified 25 ASVs with a relative abundance > 0.02% that were significantly enriched in *U. parvifolia* (Fig. 5; Supplementary Table S6). One dominant unclass. *Leotiomycetes* ASV (ASV12) accounted for approximately 15.5% of the fungal community in *U. parvifolia* and showed very strong enrichment relative to *U. glabra* (> 100-fold), representing the most prominent ASV-level difference between the two species. Additional ASVs enriched in *U. parvifolia* were affiliated with unclass. *Didymellaceae*, *Aureobasidium pullulans*, *Trichomerium* and *Lecania cyrtella*, displaying moderate to strong enrichment (5–50-fold).

Several ASVs belonging to yeast or yeast-like fungi were also significantly enriched in *U. parvifolia*, including multiple ASVs of *Filobasidium*, *Dioszegia*, *Vishniacozyma*, *Buckleyzyma aurantiaca*, *Papiliotrema*, *Knufia*, and *Taphrina*, with fold changes ranging from moderate to very strong (> 10-fold; Supplementary Table S6). In addition, ASVs affiliated with unclass. *Mycosphaerellaceae* and the chlorophyte alga *Pseudochlorella* were enriched in *U. parvifolia*. In contrast, ASVs enriched in *U. glabra* were predominantly affiliated with *Cladosporium*, *Elsinoë*, unclass. *Phaeomoniellales* and *Diaporthe cynaroidis*, often showing strong to very strong enrichment (> 10-fold), consistent with a community dominated by fewer highly abundant taxa.

### Network analysis of the bacterial and eukaryotic microbiomes

**Fig. 6:**
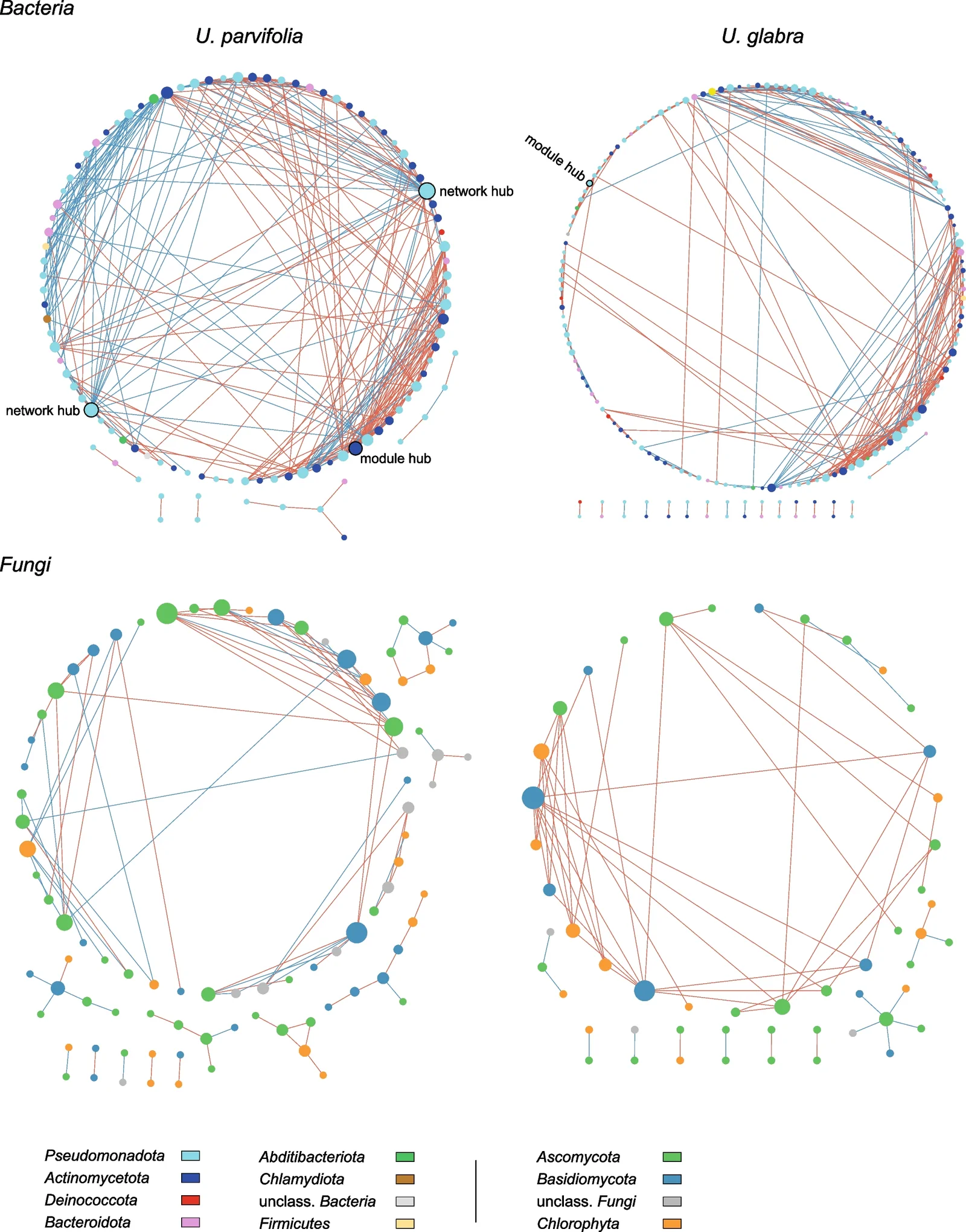
Co-occurrence network analysis of the bacterial and eukaryotic microbiomes of *U. parvifolia* and *U. glabra*. Networks were constructed using the RMT approach at the ASV level. The nodes were arranged according to detected network modules. Node size reflects node degree (number of correlations per node). In the network diagrams, red and blue links represent positive and negative associations, respectively

In a first approach, a network analysis was conducted separately at the ASV level to identify interactions between community members in each microbiome. The microbiome of *U. parvifolia* resulted in a more tightly connected bacterial network. Although the network of *U. glabra* consisted of more nodes, the number of edges (associations) was higher for *U. parvifolia* (Table 1). Accordingly, the edge-node ratio was almost twice as high and the visualized network was more complex for *U. parvifolia* (Fig. 6). Moreover, two network hubs, a module hub and 8 connectors were found for *U. parvifolia*, whereas the *U. glabra* network contained just one connector and a module hub of a rather small module (Fig. 6).

**Table 1:** Network properties of the molecular ecological network at ASV level following the RMT-based approach

Network indexes	*Bacteria*		*Fungi*	
	*U. parvifolia*	*U. glabra*	*U. parvifolia*	*U. glabra*
Total nodes	103	171	88	52
Total links—number of edges	280	258	98	61
Edge-node ratio	2.72	1.51	1.11	1.17
Maximal degree	26	13	7	10
Average degree (avgK)	5.44	3.02	2.23	2.35
Network density (D)	0.053	0.018	0.026	0.046
R square of power-law	0.805	0.917	0.957	0.952
Network hubs	2	–	–	–
Modular hubs	1	1	–	–
Connectors	8	1	–	–

When applying the same threshold value, the properties of the fungal networks were notably lower, which was also obvious in the network plot (Fig. 6). The fungal networks contained fewer nodes and edges and did not show any hubs or connectors. However, the *U. parvifolia* network was again more tightly connected (Table 1, Fig. 6).

**Fig. 7:**
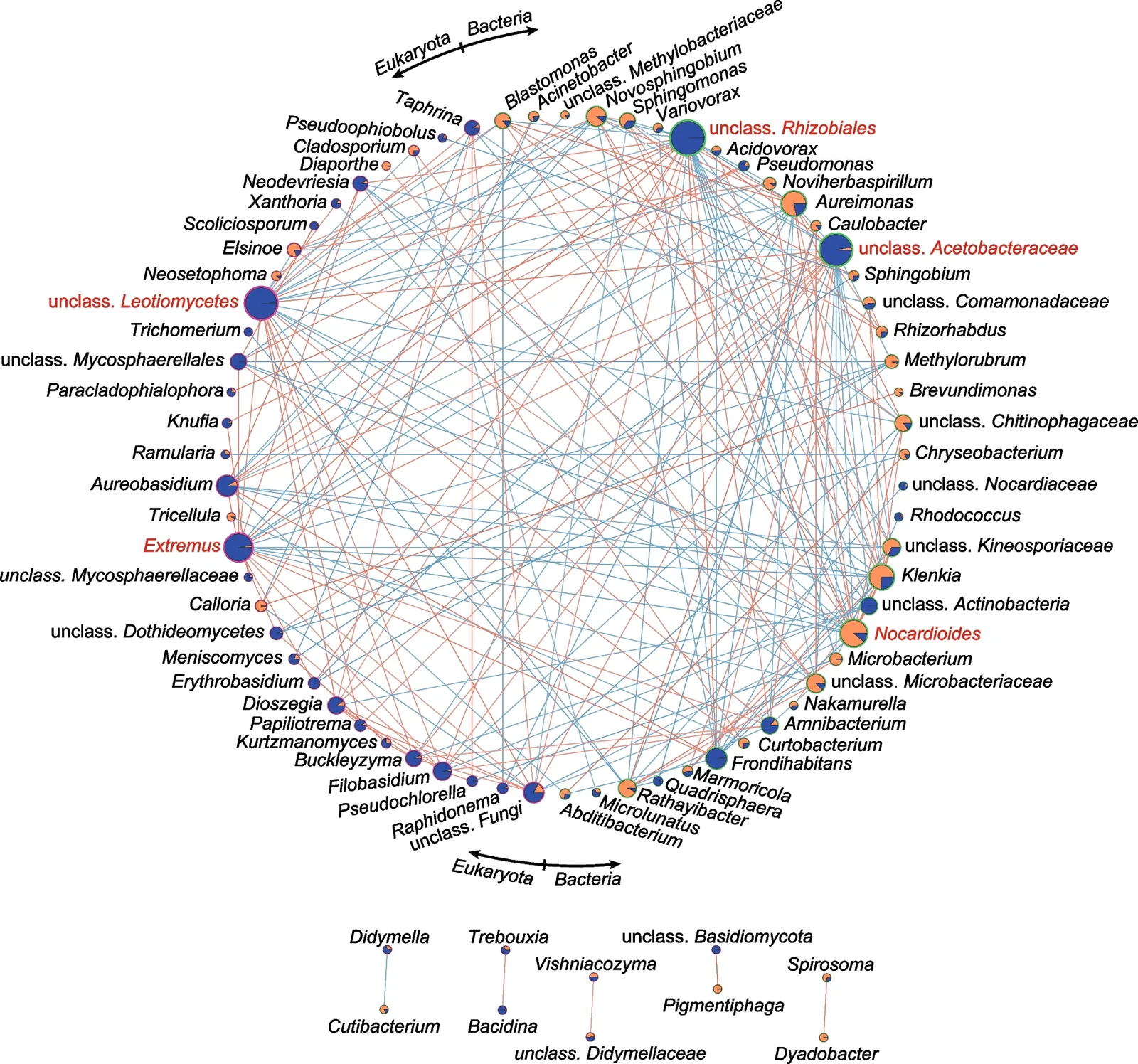
Comprehensive co-occurrence network showing significant associations among bacterial, fungal, and algal genera belonging to the microbiomes of *U. parvifolia* and *U. glabra*. The network was based on the SECOM approach at the genus level. Node size reflects the node degree (number of correlations per node). Nodes are displayed as pie charts indicating the relative abundance of each genus in *U. parvifolia* (blue) and *U. glabra* (orange). The nodes of the genera occurring with more than 80% relative abundance in only one elm species were considered as species-specific. Hub genera identified in the networks are indicated with red labels. In the network diagram, red and blue edges represent positive and negative associations, respectively (*p* < 0.001). To facilitate visualization, fungi (and algae) were arranged left and bacteria right, respectively, and sorted according to phylum. Separate associations were arranged below

A comprehensive analysis combining the bacterial and eukaryotic microbiomes of both elm species revealed a complex co-occurrence network comprising 221 significant associations (edges) among 78 genera (nodes) (Fig. 7). The network density was 0.095 and dominated by bacterial-bacterial and bacterial-fungal connections. Fungal-fungal connections were underrepresented, consistent with observations from separate networks. Network analysis identified five microbial hubs, which are nodes occupying central positions and likely exert a strong influence on community structure [44, 45]. Four hubs predominately occurred in *U. parvifolia,* including two bacterial hubs affiliated with unclass. *Rhizobiales* and unclass. *Acetobacteraceae,* and two fungal hubs affiliated with unclass*. Leotiomycetes* and *Extremus*. Collectivelly, these four hubs possessed 94 connections (48 positive, 46 negative). For *U. glabra*, only *Nocardioides* was identified as hub, with 19 connections (10 positive, 9 negative). Furthermore, the network was dominated by genera specific to each elm species: 33 nodes specific to *U. parvifolia* and 21 specific to *U. glabra* (Fig. 7). The summed degree (number of associations per node) for *U. parvifolia*-specific genera was 238, compared to 110 for *U. glabra*. Notably, 73% of the *U. parvifolia*-specific genera belong to fungi, whereas 76% of the *U. glabra*-specific genera were bacteria.

Besides the two fungal hubs, other fungal genera with strong network integration (high degree) were significantly increased in *U. parvifolia* (*Aureobasidium*, *Filobasidium*, *Dioszegia*). Since these genera are yeasts or yeast-like, the yeasts specific to *U. parvifolia* were studied in detail. In total, six highly connected yeast and yeast-like genera were often positively correlated with the bacterial hub genera unclass. *Rhizobiales* and unclass. *Acetobacteraceae* as well as the fungal hub unclass. *Leotiomycetes* (Table 2)*.* Conversely, four of the yeasts and yeast-like genera were negatively correlated with a node assigned to unclass. *Actinobacteria.* The basidiomycete yeast *Dioszegia* was positively correlated with four other highly connected yeasts (*Papiliotrema*, *Buckleyzyma*, *Filobasidium* and *Taphrina*).

**Table 2:** Positive and negative associations of highly connected yeast genera specific to *U. parvifolia*

Genus (degree)	Positive correlations	Negative correlations
*Aureobasidium*	*Frondihabitans, Pseudomonas*	*Klenkia,* unclass. *Methylobacteriaceae*
(13, 6 +, 7−)	*Neodevriesia,* unclass*. Rhizobiales,*	*Aureimonas, Novosphingobium*
	unclass*. Acetobacteraceae, Extremus*	*Nocardioides, Abditibacterium,*
		unclass. *Kineosporiaceae*
*Filobasidium*	unclass*. Rhizobiales*,* unclass. Leotiomycetes*,	*Methylorubrum, unclass. Actinobacteria*
(10, 8 +, 2−)	*Amnibacterium*,* Pseudochlorella*	
	*Dioszegia,* unclass*. Dothidiomycetes*	
	*Extremus,* unclass*. Acetobacteraceae*	
*Dioszegia*	*Papiliotrema,* unclass*. Acetobacteraceae*	*Unclass. Actinobacteria*
(9, 8 +, 1−)	unclass*. Rhizobiales*,* Buckleyzyma*	
	*Filobasidium,* unclass*. Leotiomycetes*	
	unclass*. Fungi*,* Taphrina*	
*Buckleyzyma*	*Amnibacterium, Kurtzmanomyces*	*Aureimonas,*
(7, 5 +, 2−)	unclass*. Acetobacteraceae*, unclass*. Fungi*,	unclass.* Actinobacteria*
	unclass*. Leotiomycetes*	
*Papiliotrema*	*unclass. Acetobacteraceae*, unclass.* Fungi*	
(4 +)	unclass*. Leotiomycetes*,* Dioszegia*	
*Taphrina*	*Dioszegia,* unclass*. Leotiomycetes*	*Unclass. Actinobacteria,*
(7, 4 +, 3−)	unclass* Fungi*,* Pseudochlorella*	unclass.* Chitinophagaceae, Calloria*

### Epigenetic differences at the DNA and RNA levels were confirmed for both elm species

**Fig. 8:**
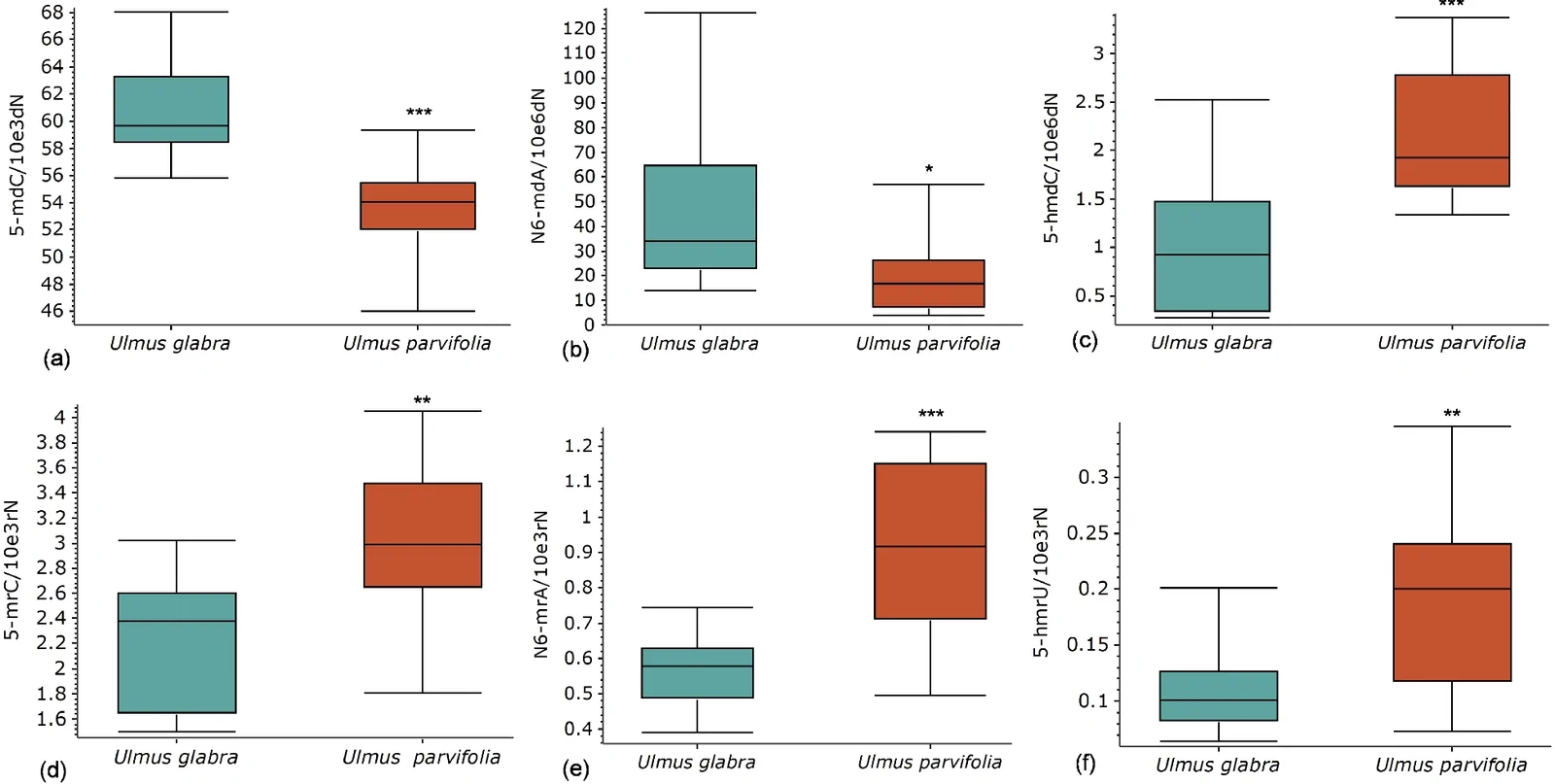
Levels of non-canonical DNA (**a** 5-mdC, **b** N6-mdA, **c** 5-hmdC) and RNA (**d** 5-mrC, **e** N6-mrA, **f** 5-hmrU) nucleosides in both elm species, measured per 10^3^ or 10^6^ nucleosides. Box plots present median, upper and lower quartiles; whiskers show minimum and maximum values, *n* = 12. Statistical data analyses: Welch’s *t*-test. Significant differences (*p* < 0.05) between both elm species are shown. *p*-values: *p < 0.05, **p < 0.01, ***p < 0.001

The presence of seven different non-canonical deoxynucleosides: 5-mdC, 5-(hydroxymethyl)−2′-deoxycytidine (5-hmdC), 5-formyl-2′-deoxycytidine (5-fdC), 5-(hydroxymethyl)−2′-deoxyuridine (5-hmdU), 2′-deoxyuridine (dU), 8-oxo-7,8 -dihydro-2′-deoxyguanosine (8-oxodG), and N6-methyl-2′-deoxyadenosine (N6-mdA) (Supplementary Table S7a) and five non-canonicalribonucleosides: 5-methylcytidine (5-mrC), N6-methyladenosine (N6-mrA), 5-hydroxymethyluridine (5-hmrU), ribothymidine (rT), and 5-hydroxymethylcytidine (5-hmrC) (Supplementary Table S7b) was established for both elm species. The deoxynucleoside 5-carboxy-2′-deoxycytidine (5-cadC) and the ribonucleoside 5-formyluridine (5-frU) were not detected in any samples. Significant differences were confirmed for the levels of some non-canonical deoxynucleosides. 5-mdC (Fig. 8a–c) was the most abundant non-canonical DNA nucleoside and 5-mrC (Fig. 8d–f) was the most abundant for RNA in both elm species. In *U. parvifolia* 53.8 5-mdC/10^3^dN were measured, which is 11.5% lower (*p* ≤ 0.001) than in *U. glabra* (60.8 5-mdC/10^3^dN), and 20.4 N6-mdA/10^6^dN which is 57.4% (*p* ≤ 0.05) lower compared to *U. glabra* (47.8 N6-mdA/10e^6^dN) (Fig. 8a, b). In contrast, 3.3 5-hmdC/10^6^dN were measured in *U. parvifolia* that is 115.06% higher (*p* ≤ 0.001) than in *U. glabra* (1.8 5-hmdC/10^6^dN) (Fig. 8c). Significant differences were also found for the levels of some non-canonical ribonucleosides between the two elm species. The mean concentrations in *U. parvifolia* of 5-mrC (3.6/10^3^dN), N6-mrA (0.9/10^6^dN), and 5-hmrU (0.2/10^6^dN) were 32.59% (*p* ≤ 0.01), 62.88% (*p* ≤ 0.001), and 32.59% (*p* ≤ 0.01) higher compared to *U. glabra,* respectively (Fig. 8d–f). The levels of the other non-canonical DNA and RNA nucleosides were not significantly different between the two elm species (Supplementary Table S7).

## Discussion

In this study, we investigated microbiome and epigenetic associated factors that may underlie the differing DED resistance observed in two elm species, *U. parvifolia* and *U. glabra*. Seedlings were co-cultivated to minimize environmental variability and to facilitate the detection of species-specific determinants. Distinct microbiome and epigenetic differences were identified between the two species, which may be associated with their contrasting stress resilience.

Comparative metabarcoding was performed on cambium and cortex tissues from surface-sterilized stems to characterize the endogenous microbiome. However, an unintended detection of a part of the epiphytic microbiome cannot be fully excluded. Additionally, lichen associations—which are not considered members of the microbiome—were detected. This may result from young microscopic lichens and lichen rhizines attached to the stem cortex. The bacterial and combined eukaryotic communities of elm stems showed significant interspecific variation in diversity, community structure, and taxonomic composition (Figs. 1, 2, and 3; Supplementary Tables S1–S4). These findings underscore the role of host genetic factors and potential seed-mediated microbial inheritance in shaping microbiome composition, thereby providing insights into the contribution of microbial communities to disease resistance and stress adaptation. Similar patterns have been reported in other plant species (review in: [46, 47]).

A key subset of microorganisms, known as the core microbiome, is widely recognized for its crucial contribution to plant health, stability, and overall fitness [48]. Both elm species showed different core microbiomes (Figs. 3 and 4). Through evolutionary processes, plants are able to selectively recruit and maintain these beneficial microorganisms, which support vital physiological processes and protect against diseases, thereby playing a fundamental role in sustaining plant well-being and resilience [48]. Therefore, identifying and comparing the core microbiomes of trees that are tolerant versus susceptible is crucial for pinpointing key microbial members that contribute to plant defense against pathogens and for understanding how microbiome interactions influence disease spread [49].

By integrating microbiomic and epigenetic analyses, our study provides a foundation for future strategies aimed at enhancing disease resilience in elms, such as microbiome optimization or targeted epigenetic modulation. This integrated perspective complements traditional genetic approaches and opens new avenues for hypothesis-driven research on host–pathogen interactions in DED, offering potential for innovative, multi-layered management strategies that have not been systematically considered to date.

### Bacterial microbiome and DED resistance

Alpha diversity was significantly higher in *U. glabra* than in the DED-resistant *U. parvifolia* (Fig. 1), suggesting a more complex but potentially less specialized microbial community in the susceptible species. In contrast, the reduced diversity observed in *U. parvifolia* may reflect stronger host filtering and functional optimization of the microbiome, a pattern frequently associated with disease-resistant plant genotypes [50, 51]. The shared Asian origin of *U. parvifolia* and *Ophiostoma* species may have facilitated long-term co-evolution, enabling the assembly of a microbiome better adapted to suppress DED-associated pathogens.

Despite identical growth conditions, PCoA and ANOSIM analyses revealed clear separation between the bacterial communities of the two elm species (Fig. 1), indicating strong host-dependent structuring. Taxa present at comparable relative abundances in both species are therefore unlikely to be primary determinants of DED resistance, directing attention toward taxa enriched in *U. parvifolia* (Fig. 2). Both microbiomes were dominated by *Pseudomonadota,* particularly *Alphaproteobacteria*, with shared genera such as *Methylobacterium*, *Sphingomonas*, and unclass. *Rhizobiales*. Although *Methylobacterium* did not differ significantly at the genus level, several ASVs within this genus were strongly associated with *U. parvifolia*, highlighting strain-level differentiation that is obscured in higher-rank analyses (Supplementary Table S5).

ASV-level patterns further support the presence of species-specific bacterial assemblages that may be associated with differences in DED susceptibility (Fig. 2; Supplementary Tables S1, S2, and S5). In *U. parvifolia*, ASVs affiliated with unclass. *Rhizobiales* represented some of the most strongly enriched taxa. Members of this order are widely implicated in plant stress tolerance and defense priming [52, 53]. Similarly, ASVs assigned to unclass. *Acetobacteraceae*—a family known for antifungal metabolite production—were consistently associated with *U. parvifolia* [54, 55]. Enrichment of *Hymenobacter* and *Quadrisphaera*, genera previously linked to stress resilience and plant-associated environments [33, 56, 57], further supports the hypothesis that the *U. parvifolia* microbiome contributes to enhanced tolerance against DED.

In contrast, *U. glabra* was characterized by enrichment of taxa such as *Klenkia*, *Nocardioides*, and members of *Microbacteriaceae*, which have been associated with plant growth promotion and general stress tolerance [58–61]. However, the susceptibility of *U. glabra* to DED suggests that these taxa alone are insufficient to confer effective protection against *O. novo-ulmi*. Notably, a greater number of abundant, species-specific taxa were associated with *U. parvifolia* than with *U. glabra* (Supplementary Tables S1 and S2), potentially reflecting stronger functional redundancy or synergistic interactions in the resistant host.

Although the two elm species shared a substantial core microbiome at the genus level, their core assemblages differed markedly in taxonomic composition (Fig. 3). Genera such as unclass. *Rhizobiales* [52], *Pseudomonas* [62], and unclass. *Acetobacteraceae* [54, 55] may contribute to DED resistance in *U. parvifolia*. These taxa are widely associated with pathogen suppression and induced resistance in plants. In *U. glabra*, other bacteria with reported resistance-promoting functions are also present in the core microbiome, including *Variovorax* [63] and members of *Chitinophagaceae* [64]; however, the continued susceptibility of this species suggests that these associations alone are insufficient to confer effective protection against DED pathogens.

### Eukaryotic microbiome composition and DED resistance

In the present study, alpha diversity of the eukaryotic microbiome and lichen associations was significantly higher in *U. parvifolia* than in *U. glabra*, in contrast to the bacterial microbiome where *U. glabra* was more diverse. Beta diversity analysis also revealed a clear separation between species, suggesting that host genetics or vertically transmitted seed microbiomes strongly influence microbial assembly (Fig. 1). Together, these patterns suggest that DED-resistant elms may host a more functionally specialized eukaryotic microbiome, potentially enriched in beneficial symbionts rather than generalist taxa, and that host genetics or vertically transmitted seed microbiomes contribute to shaping these communities.

A previous study showed that DED-resistant *U. minor* and *Ulmus pumila* genotypes exhibit a lower frequency and diversity of fungal endophytes in the xylem compared to susceptible *U. minor* genotypes [23]. However, both resistant and susceptible genotypes showed similar frequency and diversity of endophytes in the leaves and bark, indicating that resistance-associated differences in the eukaryotic microbiome may be tissue-specific.

At the phylum level, *Ascomycota* dominated the mycobiome of both elm species, consistent with previous work on *U. minor* endophytic communities [14], but the composition of core taxa differed markedly.

The core eukaryotic microbiome of *U. parvifolia* was richer and included several yeast and yeast-like taxa, such as *Aureobasidium*, *Filobasidium*, and *Dioszegia*, which are known for stress mitigation and biocontrol traits [65–70]. Yeast and yeast-like fungi can antagonize pathogens through competition for nutrients, production of lytic enzymes and secondary metabolites, emission of volatile organic compounds (VOCs), and induction of plant immune responses [68].

The dominance of several unclass. *Leotiomycetes* ASVs in *U. parvifolia* points to potentially important, yet undescribed, protective taxa. Members of *Leotiomycetes* are ecologically versatile and frequently associated with plants as endophytes or mycorrhizal partners, but the class also includes pathogens [71–75]. *Leotiomycetes* are known for producing secondary metabolites such as echinocandins, which inhibit β−1,3-glucan synthase and compromise fungal cell wall integrity [76, 77]. ASVs belonging to echinocandin-producing *Leotiomycetes* can display strong antagonistic activity against other fungi, and yeast-like forms have recently been described within this group [74, 78–80]. Therefore, the enrichment of multiple unclass. *Leotiomycetes* ASVs in *U. parvifolia* highlights a potentially diverse set of protective taxa that may contribute to disease resilience.

*Ophiostoma novo-ulmi*, the main causal agent of DED, spreads systemically in elms via blastospores in the vascular tissues, even in resistant trees [4, 81, 82]. Several yeast-like members of the core microbiome can also colonize elms systemically and may enhance resilience through hormone production and stress-alleviating compounds [14, 82]. In DED-resistant *U. minor* genotypes, taxa such as *Buckleyzyma* and members of *Trichomeriaceae* may reduce the impact of *O. novo-ulmi* through direct antagonism, VOCs production, priming of salicylic acid-dependent defenses, and improved physiological performance under stress conditions [14, 83]. The enrichment of *Buckleyzyma aurantiaca*, *Trichomerium*, and *Knufia* in *U. parvifolia* suggests similar functional roles in this elm species.

In contrast, *U. glabra* lacked strong enrichment of taxa previously associated with DED resistance. Instead, *U. glabra* harbored higher relative abundances of *Cladosporium*, *Elsinoë*, and *Phaeomoniellales*, which include stress-associated taxa as well as opportunistic or pathogenic species with limited evidence for DED protection [84–86]. The core fungi unique to *U. glabra* were dominated by two genera known to promote stress resistance, *Cladosporium* [85] and *Diaporthe* [87], both also associated with dead plant material, along with *Neoseptophoma* [88], and the pathogenic genus *Zymoseptoria* [89]. Although previous work has reported *Trichomerium*, *Aureobasidium*, *Cladosporium*, and *Vishniacozyma* as abundant leaf endophytes in *U. glabra* [90], these taxa were not enriched in stems and cambium to the same extent as in *U. parvifolia*.

Lichen-associated ASVs were also more abundant and diverse in *U. parvifolia* than in *U. glabra*. Several genera enriched in *U. parvifolia*, such as *Lecania*, *Physcia*, and *Bacidia*, are known producers of compounds with insecticidal and antifungal properties [91–95], which could contribute indirectly to DED resistance. In contrast, *Trebouxia*, the only lichen-related alga present in the core symbiotic community of both elms, occurred at similar levels in both species and is unlikely to explain differences in stress resistance.

Chlorophyte algae, particularly *Pseudochlorella*, *Raphidonema*, and *Tetratostichococcus*, were significantly enriched in *U. parvifolia*. Some chlorophyte taxa, including *Chlorella fusca*, have been shown to enhance pathogen resistance and stress tolerance in various crops [96–99]. *Pseudochlorella pringsheimii* has been associated with increased abiotic stress tolerance [100], suggesting that algal endophytes may also contribute to the improved stress resilience of *U. parvifolia*.

Taken together, *U. parvifolia* hosts a richer and functionally more diverse eukaryotic core microbiome than *U. glabra*, characterized by yeast-like fungi, lichenized taxa, and chlorophyte algae with known or plausible roles in stress mitigation and pathogen suppression. These patterns parallel observations in DED-resistant *U. minor* and support the hypothesis that a specialized eukaryotic microbiome contributes to DED tolerance in Asiatic elms, whereas *U. glabra* may lack key protective microbial partners.

### Network analysis of eukaryotic microbiomes and lichen associations

In *U. glabra* (DED-susceptible) and *U. parvifolia* (DED-resistant) seedlings, network analysis reveals critical differences in eukaryotic microbiome and lichen association patterns that align with their contrasting disease resistance phenotypes. These findings emphasize the role of microbial network complexity, cross-kingdom synergies, and hub taxa in mediating plant health and pathogen resilience.

The bacterial microbiome of *U. parvifolia* exhibited a higher edge-node ratio and greater network density compared to *U. glabra*, indicating stronger microbial interactions and functional redundancy. This aligns with studies in strawberry plants, where resistant cultivars harbor more interconnected microbial networks that enhance pathogen suppression [101, 102]. The presence of four microbial hubs (e.g., unclass. *Rhizobiales*, *Acetobacteraceae*, and unclass. *Leotiomycetes*) in *U. parvifolia* suggests these taxa act as keystone species, stabilizing the community through resource exchange or niche competition [44]. In contrast, *U. glabra*’s simpler network—dominated by fewer hubs like *Nocardioides*—reflects reduced functional coordination, potentially compromising its ability to mitigate pathogen invasion [103].

While fungal networks were less interconnected overall, *U. parvifolia*’s mycobiome showed tight integration with bacterial hubs, particularly through yeast-like fungi (*Aureobasidium*, *Filobasidium*, *Dioszegia*). These yeasts exhibited positive correlations with bacterial hubs (e.g., *Rhizobiales*) and negative correlations with *Actinobacteria*, suggesting antagonistic interactions that may suppress pathogenic taxa [104, 105]. Such cross-kingdom partnerships are critical for systemic resistance, as fungal-bacterial consortia often enhance plant immunity through metabolite exchange or niche competition. For example, *Aureobasidium pullulans* is known to prime SA-mediated defenses, while *Filobasidium* spp. produce VOCs that inhibit fungal pathogens [103].

The dominance of fungal hubs in *U. parvifolia* (73% of its specific genera) contrasts with *U. glabra*’s bacterial-dominated network (76% bacteria-specific). This suggests that DED-tolerant elms prioritize fungal recruitment to orchestrate stress responses, possibly due to superior fungal capacity for systemic colonization and secondary metabolite production [106]. Notably, the yeast genus *Dioszegia* formed a central hub with connections to multiple bacterial and fungal taxa, highlighting its role as a network stabilizer. Similar patterns have been observed in rhizosphere microbiomes of disease-resistant crops, where fungal richness enhances bacterial diversity and pathogen antagonism [101]. *Dioszegia* modulates host colonization efficiency of the white rust-causing agent *Albugo* sp. on *Arabidopsis thaliana* and significantly influences microbiome structure by acting as a keystone species, suppressing the growth and diversity of other microbes [44].

The modular structure of *U. parvifolia*’s microbiome—with tightly linked fungal-bacterial modules—mirrors findings in disease-suppressive soils, where microbial consortia intensify competition with pathogens through niche overlap [103]. For instance, the negative associations between yeasts and *Actinobacteria* in *U. parvifolia* may limit pathogen colonization by depleting shared resources or producing inhibitory metabolites [107]. Conversely, the more fragmented network in *U. glabra* may be less resilient, potentially contributing to its susceptibility to *Ophiostoma novo-ulmi*.

### Epigenetic variation at the DNA and RNA levels distinguishes both elm species

The method employed, UPLC coupled with sensitive mass spectrometry, provides a comprehensive perspective on epigenetic variation in the DNA and RNA of the holobiont. Results reflect the epigenome and epitranscriptome of both elm species alongside their microbiomes [17]. Our findings confirmed for the first time epigenetic variation at DNA and RNA levels in both elm species (Fig. 8, Supplementary Table S7).

Higher 5-mdC levels (Fig. 8a) in DNA from both species align with reports in other plants [17, 18, 108]. Significant differences in 5-mdC (*p* ≤ 0.001), N6-mdA (*p* ≤ 0.05), 5-hmdC (*p* ≤ 0.001), 5-mrC (*p* ≤ 0.01), N6-mrA (*p* ≤ 0.001), and 5-hmrU (*p* ≤ 0.01) between DED-resistant *U. parvifolia* and susceptible *U. glabra* suggest that epigenetic mechanisms may contribute to resistance and susceptibility.

Lower 5-mdC coupled with higher 5-hmdC in *U. parvifolia* may indicate a more dynamic methylation state potentially associated with enhanced responsiveness to environmental stresses, including pathogen challenge [26]. Elevated 5-hmdC levels suggest active demethylation processes, consistent with studies reporting epigenetic regulation in plant defense responses [109].

The 57.36% lower N6-mdA in *U. parvifolia* (Fig. 8b) suggests microbiome influence, as N6-mdA predominates in bacteria [110] but occurs in plants [111–113], affecting gene expression and stress adaptation [114, 115]. Debates persist on its eukaryotic role due to low levels [116]. Similarly, 62.88% higher N6-mrA (*p* ≤ 0.001), a main bacterial RNA marker, raises parallel questions [117].

Plant microbiomes influence epigenetic pathways [118], but endogenous microbiome epigenetics faces challenges like DNA separation, low biomass, and taxonomic diversity [119]. Metagenomic and single-cell epigenomics may advance this field.

5-cadC, a DNA modification typically present at low levels in mammals, was not detected, suggesting limited TET-mediated demethylation or rapid turnover in these elm species [17, 18, 108, 120]. Likewise, 5-frU, an RNA modification linked to oxidative stress, was absent, potentially reflecting low oxidative stress, efficient repair, or absent biosynthetic pathways [121]. The lack of detectable modifications does not necessarily indicate complete absence, as factors such as detection limits, physiological state, or evolutionary differences in epigenetic regulation may contribute [120]. These findings highlight the need for more sensitive detection methods and examination under stress conditions to better understand the epigenetic mechanisms underpinning stress responses and disease resistance in elms [122].

Levels of 5-mrC, N6-mrA, and 5-hmrU levels were significantly increased in *U. parvifolia* (Fig. 8d–f). Elevated 5-mrC is linked to mRNA transport, stress tolerance, and development [123–127], while higher N6-mrA enhances adaptability and viral resistance [128–130]. Other nucleosides showed no differences (Supplementary Table S7), suggesting conserved pathways. Enhanced modifications in *U. parvifolia* could contribute to DED resistance [128].

Significant differences in non-canonical nucleosides between *U. parvifolia* and *U. glabra* suggest potential adaptive advantages for *U. parvifolia* in responding to environmental challenges. Further research is needed to elucidate the functional roles of these modifications, their distribution within the holobiont, and their contributions to the species’ overall resilience.

## Conclusions

Our results confirm that the contrasting susceptibility of *U. parvifolia* and *U. glabra* to DED correlates strongly with pronounced differences in their microbial communities, lichen associations, and distinct patterns of epigenetic modifications on nucleosides. The enrichment of stress-tolerant microbiome members in *U. parvifolia*, together with its distinctive lichen associations and elevated levels of modified DNA and RNA nucleosides, could contribute to its enhanced resistance to DED. These findings provide valuable insights into the complex ecological and molecular mechanisms, including epigenetic regulation, that underpin tolerance to this devastating disease.

Understanding how epigenetic modifications influence both the host genome and its associated microbiome opens new avenues for comprehending DED resistance beyond classical genetic factors. This integrative perspective highlights the importance of considering the elm holobiont, encompassing both the host and its associated microbiome, when investigating disease resistance and may inform future elm breeding programs, conservation efforts, and disease management strategies.

Notably, many microbial taxa identified in this study have the potential to serve as keystone members in Synthetic Community (SynCom) frameworks designed to enhance DED resistance by modulating the plant microbiome. Additionally, chemical compounds produced by both microbiome constituents and associated lichens may be explored as natural deterrents to the beetle vectors that spread the disease.

By incorporating epigenetic insights into these ecological and microbial approaches, future efforts can aim to develop more resilient elm cultivars that harness genetic, epigenetic, and microbial interactions synergistically. This comprehensive strategy offers promising potential to mitigate the global impact of DED and support restoration of elm populations worldwide.

## Supplementary Information

Supplementary Material 1: Supplementary material and methods. Supplementary Fig. S1. Comparison of bacterial community structures at the genus level between *U. parvifolia* and *U. glabra* seedlings. Genera exhibiting significantly higher abundance in *U. parvifolia* are indicated in green. Supplementary Fig. S2. Comparison of the eukaryotic communities in seedlings of *U. parvifolia* and *U. glabra* at the genus level. Genera with significantly increased abundance in *U. parvifolia* are highlighted in green.

Supplementary Material 2: Supplementary Table S1. Bacteriome U. parv. Supplementary Table S2. Bacteriome U.glabra. Supplementary Table S3. Euk. microb. U.parv. Supplementary Table S4. Euk. microb. U.glabra. Supplementary Table S5. bact. ASVs. Supplementary Table S6. euk. ASVs. Supplementary Table S7. Epigenetic data.

## Data Availability

The paired sequence reads were deposited in the public repository Sequence Read Archive with the accession number PRJNA1338362.

## Abbreviations

Unclass.: Unclassified
DED: Dutch elm disease
2D-UPLC-MS/MS: Two-dimensional ultra-performance liquid chromatography with tandem mass spectrometry
5-fdC: 5-Formyl-2′-deoxycytidine
5-frU: 5-Formyluridine
rU: Uridine
5-hmdC: 5-(Hydroxymethyl)-2′-deoxycytidine
5-hmdU: 5-(Hydroxymethyl)-2′deoxyuridine
5-hmrC: 5-Hydroxymethylcytidine
5-hmrU: 5-Hydroxymethyluridine
5-mdC: 5-Methyl-2′-deoxycytidine
5-mrC: 5-Methylcytidine
8-oxodG: 8-Oxo-7,8-dihydro-2′-deoxyguanosine
dG: 2′-Deoxyguanosine
dN: Total deoxynucleosides amount
dT: 2′-Deoxythymidine
dU: 2′-Deoxyuridine
N6-mdA: N6-methyl-2′-deoxyadenosine
N6-mrA: N6-methyladenosine
UPLC: Reversed-phase ultra-performance liquid chromatography
