# Supplementary material for: Microbiome analysis and epigenetic patterns revealed distinct differences between two elm species with contrasting Dutch elm disease resistance
Source: Microbiome. 2026 Aug 19;14:204. doi: 10.1186/s40168-026-02488-5 (PMC13488114; doi:10.1186/s40168-026-02488-5)
Supplement: Supplementary file 1 [file 40168_2026_2488_MOESM1_ESM.docx]

**Supplementary Information**

**Microbiome analysis and epigenetic patterns revealed distinct differences between two elm species with contrasting Dutch elm disease resistance**

**Authors**

Hans Hoenicka^1*^, Kristina Ulrich^2*^, Charlotte Haffner^1^, Marta Starczak^3^, Daniel Gackowski^3^, Ben Bubner^2^, Andreas Ulrich^4^

^1^ Thünen-Institute of Forest Genetics, Sieker Landstr. 2, 22927 Großhansdorf, Germany

^2^ Thünen Institute of Forest Genetics, Eberswalder Chaussee 3a, 15377 Waldsieversdorf, Germany

^3^ Department of Clinical Biochemistry, Faculty of Pharmacy, Collegium Medicum in Bydgoszcz, Nicolaus Copernicus University in Toruń, Karlowicza 24, 85-095 Bydgoszcz, Poland.

^4^ The Leibniz Centre for Agricultural Landscape Research (ZALF), Eberswalder Straße 84, 15374 Müncheberg, Germany

Corresponding author (h.hoenicka@thuenen.de)

*These authors contributed equally to this work

ORCIDs: Hans Hoenicka: https://orcid.org/0000-0001-7226-3772, Daniel Gackowski: https://orcid.org/0000-0002-7259-1511, Marta Starczak: https://orcid.org/0000-0003-4098-7412

**Supplementary Material and Methods**

Plant substrate composition

**Fine-structured substrate**

Composition (v/v unless otherwise stated):

* White peat: 60%

* Black peat: 20%

* Perlite (0–3 mm): 20%

Additives (per m³):

* Sand: 100 kg

* Radigen (trace element mixture): 100 g

* Wetting agent: 900 mL

Target pH: 5.0

**Container substrate with clay**

Composition (v/v unless otherwise stated, per EN m³ [European standard cubic meter]):

* White peat: 60%

* Mixed peat: 20%

* Wood fiber: 15%

* Cocopeat: 5%

Additives (per m³):

* Moist clay: 75 kg

* PG-Mix (NPK fertilizer, 14-16-18): 1.2 kg

* Radigen (trace element mixture): 50 g

* Wetting agent: 500 mL

Target pH: 5.8

Structure: coarse

Sample Sterilization and Preparation for DNA Extraction

1. Remove samples from the −20 °C freezer and select the required amount of material for sterilization.
2. Transfer material to a 15 mL Falcon tube.
3. Add sterile deionized water to cover the material completely. Incubate for 5 min, shake briefly, and remove water using a filter-tip pipette or by decanting.
4. Repeat step 3.
5. Add 70% non-denatured ethanol and incubate for 30–60 s with brief shaking.
6. Immediately remove ethanol and rinse with sterile deionized water for 5 min with brief shaking.
7. Add 0.5% sodium hypochlorite solution and incubate for 3 min with brief shaking.
8. Immediately remove sodium hypochlorite and rinse with sterile deionized water for 5 min with brief shaking.
9. Rinse again with sterile deionized water for 5 min with brief shaking.
10. Cut cambium (preferably without bark, although this can be challenging) on baked glass Petri dishes. Use a new dish and scalpel blade for each sample. Sterilize other instruments (pipettes, scissors) at 250 °C between samples.
11. Place the isolated tissue in labeled Eppendorf tubes, freeze in liquid nitrogen, and store at −20 °C or grind immediately for DNA extraction.
12. Include a negative control with every extraction.
13. If needed, elute twice with 50 µL per sample into separate Eppendorf tubes; pool material from duplicate samples as required.

 **Supplementary Results**

**Fig. S1**	Comparison of bacterial community structures at the genus level between *U. parvifolia* and *U. glabra* seedlings. Genera exhibiting significantly higher abundance in *U. parvifolia* are indicated in green.

**Fig. S2** Comparison of the eukaryotic communities in seedlings of *U. parvifolia* and *U. glabra*, at the genus level. Genera with significantly increased abundance in *U. parvifolia* are highlighted in green.
